# A Hybrid Risk Factor Evaluation Scheme for Metabolic Syndrome and Stage 3 Chronic Kidney Disease Based on Multiple Machine Learning Techniques

**DOI:** 10.3390/healthcare10122496

**Published:** 2022-12-09

**Authors:** Mao-Jhen Jhou, Ming-Shu Chen, Tian-Shyug Lee, Chih-Te Yang, Yen-Ling Chiu, Chi-Jie Lu

**Affiliations:** 1 Graduate Institute of Business Administration, Fu Jen Catholic University, New Taipei City 242062, Taiwan; 2 Department of Healthcare Administration, College of Healthcare & Management, Asia Eastern University of Science and Technology, New Taipei City 220303, Taiwan; 3 Artificial Intelligence Development Center, Fu Jen Catholic University, New Taipei City 242062, Taiwan; 4 Department of Business Administration, Tamkang University, New Taipei City 251301, Taiwan; 5 Department of Medical Research, Department of Medicine, Far Eastern Memorial Hospital, New Taipei City 22056, Taiwan; 6 Graduate Institute of Clinical Medicine, National Taiwan University College of Medicine, Taipei 10002, Taiwan; 7 Graduate Institute of Medicine and Graduate Program of Biomedical Informatics, Yuan Ze University, Taoyuan 32003, Taiwan; 8 Department of Information Management, Fu Jen Catholic University, New Taipei City 242062, Taiwan

**Keywords:** machine learning (ML), Metabolic syndrome (MetS), chronic kidney disease (CKD), end-stage kidney disease (ESKD), hybrid risk factor

## Abstract

With the rapid development of medicine and technology, machine learning (ML) techniques are extensively applied to medical informatics and the suboptimal health field to identify critical predictor variables and risk factors. Metabolic syndrome (MetS) and chronic kidney disease (CKD) are important risk factors for many comorbidities and complications. Existing studies that utilize different statistical or ML algorithms to perform CKD data analysis mostly analyze the early-stage subjects directly, but few studies have discussed the predictive models and important risk factors for the stage-III CKD high-risk health screening population. The middle stages 3a and 3b of CKD indicate moderate renal failure. This study aims to construct an effective hybrid important risk factor evaluation scheme for subjects with MetS and CKD stages III based on ML predictive models. The six well-known ML techniques, namely random forest (RF), logistic regression (LGR), multivariate adaptive regression splines (MARS), extreme gradient boosting (XGBoost), gradient boosting with categorical features support (CatBoost), and a light gradient boosting machine (LightGBM), were used in the proposed scheme. The data were sourced from the Taiwan health examination indicators and the questionnaire responses of 71,108 members between 2005 and 2017. In total, 375 stage 3a CKD and 50 CKD stage 3b CKD patients were enrolled, and 33 different variables were used to evaluate potential risk factors. Based on the results, the top five important variables, namely BUN, SBP, Right Intraocular Pressure (R-IOP), RBCs, and T-Cho/HDL-C (C/H), were identified as significant variables for evaluating the subjects with MetS and CKD stage 3a or 3b.

## 1. Introduction

Suboptimal health status is a dynamic and intermediate bodily condition between health and disease. Various indicators of suboptimal health must be considered during the prevention of chronic diseases to achieve better health protection. Metabolic syndrome (MetS) is a collection of suboptimal health risk indicators. According to the definition provided by the Health Promotion Administration (HPA) and Ministry of Health and Welfare (MOHW) [1], the five diagnostic criteria of MetS are excessive abdominal fat, high blood pressure, high fasting plasma glucose levels, high fasting triglycerides levels, and elevated high-density lipoprotein-cholesterol levels. A person who meets three or more of these criteria is diagnosed with MetS [1]. The accumulation of MetS risk factors increases the risk of chronic kidney disease (CKD) and other major chronic diseases [2,3,4].

CKD refers to the irreversible and progressive loss of kidney function caused by prolonged damage to the renal tissue for several months or years. According to the American Kidney Foundation’s definition of CKD, the disease consists of the following stages: 1, 2, 3a, 3b, 4, and 5 [5,6], each of which is defined in Table 1. The middle stages, 3a and 3b, indicate moderate renal failure. Patients in CKD stage 3a are in the final stage of early CKD, while those in CKD stage 3b are in the earliest stage of end-stage kidney disease (ESKD). Thus, stage 3b is a critical point of renal function deterioration as it requires kidney dialysis. Patients in stages 3a and 3b exhibit differences in their morality risk [7,8].

MetS and CKD are important risk factors for many comorbidities and complications [9,10]. Much research has denoted a positive correlation between MetS and CKD [11,12]. Furthermore, MetS diagnosis is an effective predictor of CKD [12]. Studies on CKD prediction have identified four major risk factors: demographic variables (e.g., age, education level), anthropometric parameters (e.g., body mass index, body fat), blood examination indicators (e.g., blood urea nitrogen, uric acid), and lifestyle habits (e.g., smoking status, alcohol consumption) [13,14,15,16]. Thus, they are often used in many studies to construct CKD analytical models through machine learning (ML)-based data analysis methods [16,17,18,19,20].

ML techniques are extensively applied in numerous studies on medical informatics and suboptimal health status [3,16,21,22,23,24]. They are often used to identify critical predictor variables or risk factors as they can effectively investigate the complex relationships between risk factors and outcomes, based on their promising predictive performance with vast amounts of medical data [16,22,23,25].

Because some ML techniques can identify important predictor variables, a single technique for selecting important predictor variables and risk factors may result in a localized optimal risk that generates a single ranking of the variables. A variable ensemble is often used to integrate the different variables that are selected [26]. Relevant studies have also demonstrated that using variable ensembles improves the robustness of the selected variables, compared to a single variable selection technique, and reduces the bias and variance of the results [27,28,29,30].

Existing studies that utilize ML methods to perform CKD data analysis mostly analyze the patients directly [16,17,20,31,32,33,34], and few studies have discussed the predictive models and important risk factors for CKD patients with MetS. Several studies have constructed predictive models for MetS patients, as well as their risk factors. Although patients in stages 3a and 3b of CKD vary in disease progression and mortality risk [35,36], they share highly similar clinical presentations. Thus, this study aims to examine the ML predictive models and important risk factors for CKD stages 3a and 3b patients with MetS by using ML techniques.

This study aims to construct an effective hybrid important risk factor evaluation scheme for CKD stages 3a and 3b patients with MetS, based on ML predictive models. Our study used six well-known and effective ML techniques—random forest (RF), logistic regression (LGR), multivariate adaptive regression splines (MARS), extreme gradient boosting (XGBoost), gradient boosting with categorical features support (CatBoost), and light gradient boosting machine (LightGBM)—to develop ML predictive models [16,18,19,37,38,39]. The important risk factors identification results can provide valuable information regarding the prevention of CKD and health promotion.

The rest of this paper is organized as follows: Section 2 describes the used materials and the proposed scheme. Section 3 presents the experiment results. Section 4 discusses the findings of the study. Finally, the study is concluded in Section 5.

## 2. Materials and Methods

### 2.1. Data

This study has selected a large database of sub-health groups in Taiwan, the MJ Health Checkup-based Population Database (MJPD, http://www.mjhrf.org/main/page/resource/en/#resource07, accessed on 1 August 2022). It has published more than dozens of international journal papers, including 2 JAMA and 6 Lancet journal papers. This study was approved by the institutional review board of Far Eastern Memorial Hospital (FEMH-IRB) (No:_IRB-110027-E Approved Date: 15 February 2022) and the MJ Health Research Foundation, and registered on ClinicalTrials.gov (ID: NCT05225454).

**Figure 1:**
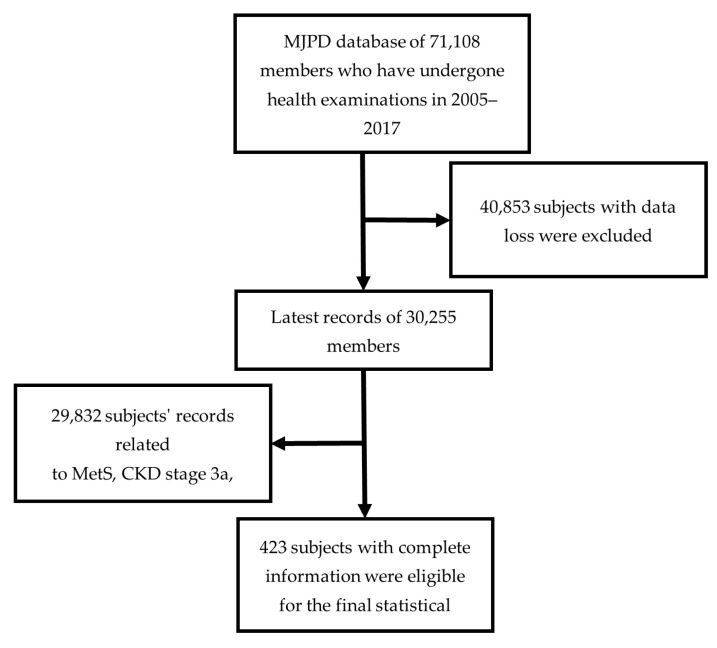
Subject identification process.

Figure 1 shows the all-subjects identification process, and the complete data were collected from the MJPD. A total of 71,108 members from 2005 to 2017 comprised the health examination indicators and questionnaire responses. Table 2 shows the 34 health examination indicators and questionnaire variables. Among the 34 variables, CKD is the target variable and the other 33 indicators are predictor variables. Given that each member might have multiple examination records, those who had undergone multiple health examinations only had their latest records analyzed. In addition, subjects whose data had missing variables were excluded. After data processing, 30,255 subjects were eligible. We applied the MOHW’s references and definitions of MetS and CKD to identify 423 MetS patients who were also diagnosed with CKD stages 3a or 3b. Table 3 presents the statistical analysis results of the participants’ demographic data. A total of 375 patients (88.65%) were diagnosed with CKD stage 3a, while the remaining had CKD stage 3b.

### 2.2. Proposed Hybrid Risk Factor Evaluation Scheme

**Figure 2:**
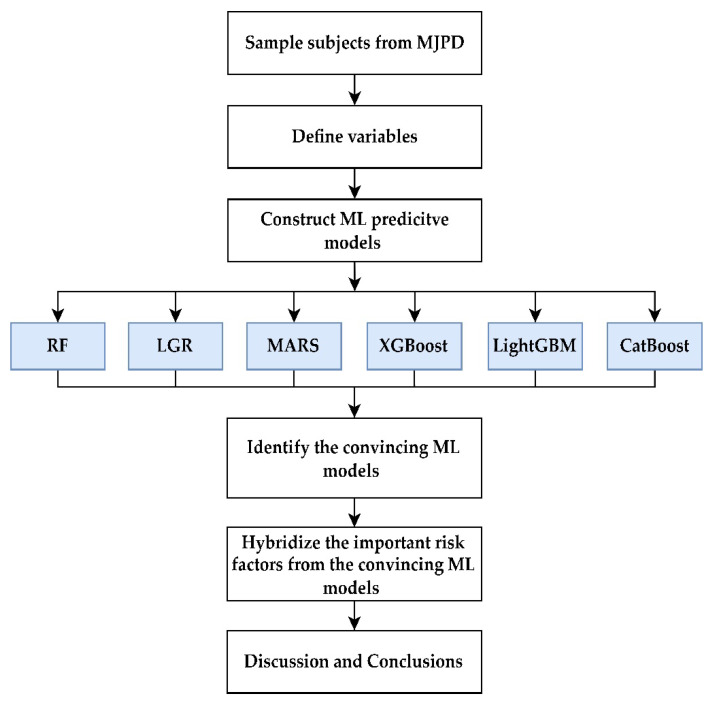
Proposed hybrid risk factor evaluation scheme.

On the basis of the six ML methods, including RF, LGR, MARS, XGBoost, CatBoost, and LightGBM, this study developed a hybrid important risk factor identification scheme for the subjects with MetS and CKD stage 3a or 3b. The six ML methods used are based on different concepts and characteristics to develop the classification models [40,41,42,43,44,45]. RF, XGBoost, CatBoost, and LightGBM are tree-based algorithms. LGR and MARS are non-parametric methods. Since they are based on different characteristics to construct effective algorithms and identify important risk factors for medical data analysis, the important variables identification results of the six methods are integrated to provide more stable and robust results. Figure 2 shows the proposed hybrid risk factor evaluation scheme.

As shown in Figure 2, the first step was to sample the MetS subjects who were diagnosed with CKD stage 3a or 3b from through the MJPD health examination database. Next, we defined the predictor variables and target variable. We used 33 risk factors as our predictor variables and CKD as the target variable. After consolidating the data, we built the RF, LGR, MARS, XGBoost, CatBoost, and LightGBM predictive models.

RF is a decision tree approach based on ensemble technology [40]. Its principle is to construct several unpruned decision trees, aggregate all the trees into a forest, and then generate the final model by taking the majority vote or average value of the trees. LGR is the typically most used ML method that generalizes linear models with canonical link functions [41]. Its aim is to minimize the relative cost function using a logistic function and perform model fitting using a maximum likelihood function.

MARS is a nonparametric and nonlinear statistical method in which several linear segments with different gradients are used to automatically examine the nonlinearity and dependency between multidimensional input and output variables, and then generate the final optimum nonlinear prediction model [42]. XGBoost is a decision tree-based approach that applies gradient boosting to generate multiple weak models. When each weak model is generated, the defects or shortcomings of the previous model are corrected. Finally, accuracy categorization is achieved by aggregating all the generated weak models [43].

LightGBM is a decision tree-based distributed gradient boosting framework that utilizes advanced histograms. In an iteration, it learns the approximate value of decision tree residuals based on one-side sampling and negative gradient fitting [44]. CatBoost is a gradient-boosting decision tree technique in which sequential boosting methods are combined with gradient boosting and multiple categorical features [45]. In CatBoost, the tree combinations and categorical features generated through gradient boosting are aggregated into a sequence to generate the final model.

For constructing each ML model, we randomly divided the whole dataset into 80% training data set and 20% testing dataset. The ten-fold cross validation (CV) method was used to perform hyperparameter tuning. The selected final model is the model of the best hyperparameter configuration. This process was performed ten times.

Balanced accuracy (BA), sensitivity, specificity, and area under the receiver operating characteristic (ROC) curve (AUC), are four well-known metrics [46,47,48] utilized to assess the six ML models’ performance. To identify the convincing ML models, the widely used LGR was viewed as the baseline model in this study. The ML model’s performance that is greater than or equal to that of the LGR model is considered as the convincing model.

To rank the importance of each predictor variable, we applied the “caret” R package of version 6.0-90 [49] to each of the six methods to produce each variable’s importance value. In each model, the most important predictor variable is set as ranking 1, whereas the least important predictor variable is defined as 33, because we used 33 predictor variables in this study. Different ML methods may produce different importance rankings for each predictor variable, due to their different specific characteristics. To obtain more stable and integrable ranking results, we hybridized the importance of each variable by averaging its ranking values from the convincing ML models.

In the last step, the research findings regarding the identified important risk factors were discussed to present the conclusions of our study.

This study utilized the RStudio of version 1.1.453 and R programming language of version 3.6.2 for modeling (http://www.R-project.org, accessed on 1 September 2022; https://www.rstudio.com/products/rstudio/, accessed on 1 September 2022). In addition, each model was constructed using an R-based software package. Model construction in RF, LGR, MARS, XGBoost, CatBoost, and LightGBM was through randomForest version 4.7-1.1 [50], stats version 3.6.2, earth version 5.3.1 [51], XGBoost version 1.6.0.1 [52], catboost version 0.25.1 [53], and lightgbm version 3.3.2 [54], respectively. During the model construction process, the best hyperparameter was found using caret version 6.0-93 [49].

## 3. Results

**Figure 3:**
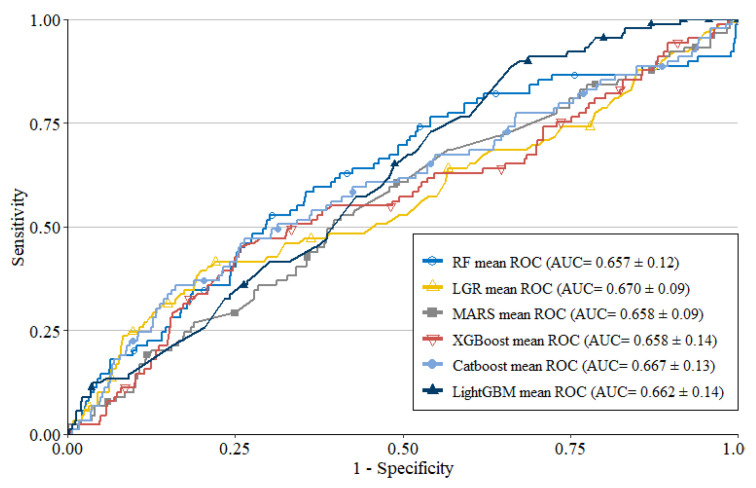
ROC curves of the six methods.

This study applied six ML techniques, including RF, LGR, MARS, XGBoost, CatBoost, and LightGBM, to build predictive models for patients with MetS and CKD stage 3. Table 4 depicts the mean prediction performances of the six models after ten learning cycles, as well as the means and standard deviations (SDs) of the four performance metrics used. Figure 3 demonstrates the ROC curves of the six models. From Table 4, it can be observed that the prediction performances of the six models were similar, and the AUC of each model was greater than 0.657. The LGR has the highest AUC value of 0.670 and the RF has the lowest AUC value of 0.657.

To evaluate the performance of the six methods, DeLong’s test was used since it is one of the effective tests employed to evaluate the statistically significant difference between two models’ AUC values [55]. We used DeLong’s test to compare AUC values between the model with the highest AUC model (i.e., LGR model in this study) to each of the remaining five ML methods. Table 5 depicts the results of DeLong’s test. It can be determined from the table that the performance difference between the LGR model and each ML method is not significant, since all *p*-values are greater than 0.05. Therefore, the six models’ prediction performances were alike and can be viewed as the convincing models. However, it is still worth noting that, from Table 4, the LGR was relatively the best ML model in this study because it can generate the highest mean balanced accuracy, specificity, and AUC values of 0.719, 0.761, and 0.670, respectively.

Because the six methods used are all considered to be convincing models, we used the variable importance generated by all six methods as the basis for our risk factor ensemble.

Table 6 shows the overall importance ranking of each predictor variable based on the six convincing models. Note that only the first 15 variables of Table 2 are shown. The “Average ranking of RF” to “Average ranking of LightGBM” are the average rankings, with the modeling of each of the six models repeated ten times. The different models produced different variable importance ranking results based on their modeling rules. In order to hybridize the findings of the six models, we summarized the ranking results of the six models equally in the proposed scheme. We obtained the “Average ranking of the six models” with simple averaging values from the six models.

**Figure 4:**
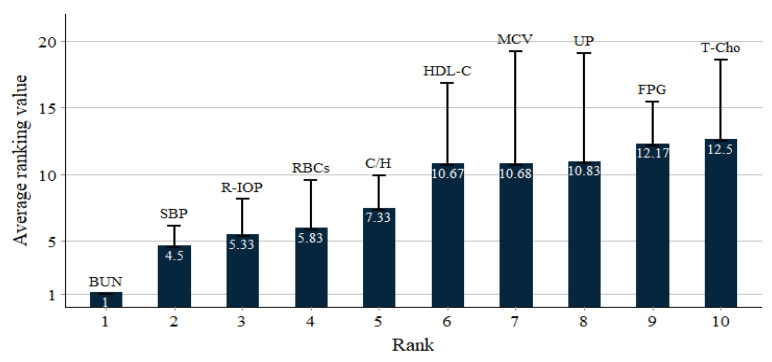
The ranked top ten most important variables.

To clarify the ranking, Figure 4 shows the ranked top ten important variables by increasing order of the average ranking values of the six models. From Figure 4, to compactly discuss the important predictor variables, based on physicians’ recommendations, the top five important predictor variables, namely BUN, SBP, R-IOP, RBCs and C/H, were identified as significant variables for assessing the subjects with MetS and CKD stage 3a or 3b.

## 4. Discussion

Most of the previous academic literature has confirmed the related risk factors of CKD, including sex, age, race, obesity, smoking, unhealthy diets, family-related history, proteinuria, and anemia. It is associated with chronic diseases including metabolic syndrome, type 2 diabetes, hypertension, cardiovascular disease, hyperlipidemia, hyperuricemia, etc., and related indicators were also found to correlate with CKD. Based on the results of previous studies, many important risk factors of CKD, such as BUN, creatinine (Cr.), UA, SBP, DBP, WC, BMI, BF, FPG, T-Cho, and LDL were known [16,17,56,57,58,59,60], in addition to indicators such as SGPT, SGOT [16], Education [16,56], RBCs, UP (Urine Protein) [17].

In recent years, three related studies have been published that use different analytical tools and subjects to determine the risk factors for CKD in Taiwan [16,17,59]. Chang et al. (2020) consulted the Elderly Health Examination Database and used 2006–2012 data from 297,603 elderly people aged 65 years and older in Taipei City, Taiwan. Employing the non-CKD criteria with the G1 and G2 stages (e-GFR > 60 mL/min/1.73 m^2^), their results showed a 29.7% e-GFR reduction in the likelihood of CKD diagnosis. The study found smoking to be significantly associated with an elevated risk of reduced e-GFR, and found physical exercise and healthy lifestyle habits to be significantly associated with increased e-GFR. Additionally, it found CVD, hypertension, obesity, and diabetes-related indicators to be linked to an increased risk of developing CKD [59]. Another study published in China used the same criteria (e-GFR > 60 mL/min/1.73 m^2^) to detect CKD among 15,229 subjects (mean age: 62.8 years) from the Dongfeng–Tongji examination dataset (2008–2013). It found that BMI and MetS are potential indicators of CKD risk among elderly people [60].

Shih et al. (2020) analyzed data from an adult health examination dataset, as well as data on elderly adults they collected from three physical examination centers and 32 clinics in Taiwan (2015–2019). However, this study features a notable limitation: the G2 stage was not rigorous when it was used to represent and indicate CKD subjects. It was selected out of 14,169 non-CKD subjects (63.37 ± 11.56 years) and 5101 CKD subjects (69.19 ± 10.74 years)—a total of 19,270 subjects—with effective records, but they determined CKD by using the G1 stage (e-GFR ≥ 90 mL/min/1.73 m^2^) to indicate non-CKD. The study found the UP-Cr. ratio, proteinuria (PRO), RBCs, FPG, TG, T-Cho, age, and gender to be important risk factors for early CKD prediction [17]. Interestingly, they identified RBCs, in addition to UP, as an important factor, though they did not elaborate on it. Previous research on UP features supports data on the correlation with RBCs; in fact, some studies show that it may be a risk factor for hypertension [48].

This study is the follow-up research to Chiu et al.’s (2021) study. The datasets were collected from four major health screening centers in the northern, central, and southern parts of Taiwan (2010–2015). A total of 65,394 subjects were included in the MJPD database for the analysis of 18 risk indicators, CKD was determined by using the criteria with the G2 stages (e-GFR > 60 mL/min/1.73 m^2^). The MJPD datasets were of the sub-health population, including more young subjects, aged around 30 to 50 years old (y/o). The study results showed that BUN and UA were identified as the first and second most important indicators, and SBP, SGPT, SGOT, and LDL-C were also related risk factors. Interestingly, socioeconomic status (SES)-related education was found to be the third important indicator in this study [16].

From the perspective of preventive medicine, the knowledge of risk factors facilitates early detection and, in turn, allows for targeting and improving relevant lifestyle habits, enabling people to avoid serious chronic diseases. In this study, we continued to use MJPD datasets [16], though notably with a younger sample. However, unlike the three most prominent previous CKD-related studies in Taiwan [16,57,59], we raised the criteria for CKD, asserting that CKD is stage 3b in the earliest stage of end-stage renal disease (e-GFR > 45 mL/min/1.73 m^2^). At the same time, we increased the number of data-covered years (2005–2017) to increase the sample size. The MJPD dataset excluded the subjects’ records related to anything but MetS, CKD stage 3a, and CKD stage 3b. Out of a total of 423 subjects, 88.65% were diagnosed with stage 3a CKD, and 11.35% were diagnosed with stage 3b CKD. BUN, SBP, R-IOP, RBCs, and C/H were identified as the five most important variables for evaluating subjects with MetS, CKD stage 3a, and CKD stage 3b.

### Limitations

In order to add the variables found in related studies and the variables that the researcher is interested in, and because analyzing too many research variables may affect the Area Under the Curve (AUC) of the algorithm, it is recommended that follow-up studies appropriately reduce variable analysis, or integrate more relevant variables, such as L-IOP and R-IOP, or T-Cho, HDL-C, LDL-C, and C/H related indicators. In addition, for a smaller number of samples, follow-up research may be able to further advance the analysis of the two risk factor values of relative importance value (RIV) or ordinal ranking value (ORV).

## 5. Conclusions

This study proposed innovative algorithms for the analysis of health-screening data pertaining to the third stage of CKD: the earliest stage of ESKD. This study contributed 33 relevant research variables, including R-IOP, RBCs, and T-CHO/HDL-C, outlining their varied associations with risk indicators identified in previous studies. This study suggested that some factorial combinations could potentially be used to separate individuals with stage 3a CKD from those with stage 3b CKD, facilitating the design of prospective studies in the future. We believe that this study has made several valuable contributions to the literature, including some that will aid in the prevention and treatment of CKD and the evaluation of high-risk groups in the third stage.

## Figures and Tables

**Table 1:** Stages of CKD.

Stage	1	2	3a	3b	4	5
**e-GFR value**	≥90	89–60	59–45	45–30	30–15	<15 or dialysis
**Description**	Kidney damage with normal or e -GFR	Kidney damage with normal or mild e-GFR	Mild-moderately decreased e-GFR	Moderately-severely decreased e-GFR	Severely decreased e-GFR	Severe Renal failure

**Table 2:** Variables for predicting the subjects with MetS and CKD stage 3a or 3b.

Abbreviation	Variables	Unit/Description
BMI	Body Mass Index	kg/m^2^
BF	Body Fat	% (@TANITA, DC-430MA)
WC	Waist Circumference	cm
SBP	Systolic Blood Pressure	mmHg
DBP	Diastolic Blood Pressure	mmHg
FPG	Fasting Plasma Glucose	mg/dL
L-IOP ^#^	Left Intraocular Pressure	mmHg
R-IOP ^#^	Right Intraocular Pressure	mmHg
r-GT	Gamma Glutamyl Transpeptidase	U/L
BUN	Blood Urea Nitrogen	mg/dL
UA	Uric Acid	mg/dL
TG	Triglyceride	mg/dL
T-Cho	Total Cholesterol	mg/dl
HDL-C	High Density Lipoprotein-Cholesterol	mg/dL
LDL-C	Low Density Lipoprotein-Cholesterol	mg/dL
C/H	T-Cho/HDL-C	the vascular risk predicts index
MS	Marital Status	(1) Single, (2) married, remarried, cohabiting, (3) divorced, (4) widowed
EL	Educational Level	(1) No formal education, (2) elementary school, (3) secondary school, (4) high school, (5) college, (6) university, (7) graduate school
FI	Yearly Family Income	(1) Unwaged, (2) NTD ≤ 200,000, (3) NTD 200,001–400,000, (4) NTD 400,001–800,000, (5) NTD 800,001–1,200,000, (6) NTD 1,200,001–1,600,000, (7) NTD 1,600,001–2,000,000; NTD: New Taiwan Dollar.
HC	Hip Circumference	cm
WHR	Waist–hip Ratio	%
LEE	Lower Extremity Edema	(1) No, (2) Yes
SGOT	Serum Glutamic-Oxaloacetic Transaminase	U/L
SGPT	Serum Glutamic-Pyruvic Transaminase	U/L
BMD	Bone Mass Density	Dual energy X-ray Absorptiometry (DEXA)
RBCs	Red Blood Cells	10^6^/μl
Hb	Hemoglobin	g/dl
MCV	Mean Cell Volume	fl
UP	Urine Protein	Qualitative test, (1) non, (2) +−, (3) + (4) ++, (5) +++, (6) ++++
GU	Glucose Urine	Qualitative test, (1) non, (2) +−, (3) + (4) ++, (5) +++, (6) ++++
CS	Current Smokers	(1) Never, (2) passive smoking, (3) quit, (4) occasional, (5) addicted
AD	Alcohol Drinkers	(1) Never, (2) quit, (3) 1–2 times a week, (4) 3–4 times a week, (5) 5–6 times a week, (6) addicted
CBN	Chewing “Betel Nut”/“Areca catechu”	(1) Never, (2) quit, (3) 1–3 times a week, (4) 4–5 times a week, (5) addicted
CKD	Chronic Kidney Disease	(1) CKD stage 3a, (2) CKD stage 3b

Note: #, Intraocular pressure was measured in this database as individuals received the measurement during health check-ups.

**Table 3:** Subject Demographics.

Variables		Mean ± SD	Variables		*n* (%)
BMI		27.19 ± 3.29	FI	(1) Unwaged	78 (18.44%)
BF		30.32 ± 6.99		(2) NTD ≤ 200,000	60 (14.18%)
WC		90.21 ± 8.50		(3) NTD 200,001–400,000	87 (20.57%)
SBP		138.18 ± 20.54		(4) NTD 400,001–800,000	82 (19.39%)
DBP		84.30 ± 12.50		(5) NTD 800,001–1,200,000	47 (11.11%)
FPG		123.20 ± 34.35		(6) NTD 1,200,001–1,600,000	35 (8.27%)
L-IOP		14.60 ± 3.40		(7) NTD 1,600,001–2,000,000	34 (8.04%)
R-IOP		14.51 ± 3.28	LEE	(1) No	418 (98.82%)
r-GT		38.95 ± 45.14		(2) Yes	5 (1.18%)
BUN		18.54 ± 4.74	UP	(1) non	333 (78.72%)
UA		7.43 ± 1.64		(2) +−	34 (8.04%)
TG		194.49 ± 78.31		(3) +	29 (6.86%)
T-Cho		204.55 ± 37.69		(4) ++	16 (3.78%)
HDL-C		47.36 ± 9.98		(5) +++	11 (2.60%)
LDL-C		123.30 ± 34.39		(6) ++++	NA
C/H		4.42 ± 0.85	GU	(1) non	405 (95.74%)
HC		99.28 ± 6.22		(2) +−	7 (1.65%)
WHR		0.91 ± 0.06		(3) +	3 (0.71%)
SGOT		28.61 ± 13.88		(4) ++	2 (0.47%)
SGPT		34.19 ± 21.16		(5) +++	6 (1.42%)
BMD		0.35 ± 1.43		(6) ++++	NA
RBCs		4.84 ± 0.54	CS	(1) Never	292 (69.03%)
Hb		14.48 ± 1.47		(2) Passive smoking	15 (3.55%)
MCV		43.19 ± 4.34		(3) Quit	57 (13.48%)
UR		1.1 ± 0.55		(4) Occasional	13 (3.07%)
				(5) Addicted	46 (10.87%)
**Variables**		***n* (%)**	AD	(1) Never	327 (77.3%)
MS	(1) Single	12 (2.84%)		(2) Quit	22 (5.2%)
	(2) Married, remarried, cohabiting	332 (78.49%)		(3) 1–2 times a week	44 (10.4%)
	(3) Divorced	13 (3.07%)		(4) 3–4 times a week	17 (4.02%)
	(4) Widowed	66 (15.60%)		(5) 5–6 times a week	NA
EL	(1) No formal education	30 (7.09%)		(6) Addicted	13 (3.07%)
	(2) Elementary school	101 (23.88%)	CBN	(1) Never	376 (88.89%)
	(3) Secondary school	51 (12.06%)		(2) Quit	36 (8.51%)
	(4) High school	64 (15.13%)		(3) 1–3 times a week	3 (0.71%)
	(5) College	55 (13.00%)		(4) 4–5 times a week	3 (0.71%)
	(6) University	74 (17.49%)		(5) Addicted	5 (1.18%)
	(7) Graduate school	48 (11.35%)	CKD	(1) CKD stage 3a	375 (88.65%)
				(2) CKD stage 3b	48 (11.35%)

Note: BMI, body mass index; BF, body fat; WC, waist circumference; SBP, systolic blood pressure; DBP, diastolic blood pressure; FPG, fasting plasma glucose; L-IOP, left intraocular pressure; R-IOP, right intraocular pressure; r-GT, gamma glutamyl transpeptidase; BUN, blood urea nitrogen; UA, uric acid; TG, triglyceride; T-Cho, total cholesterol; HDL-C, high density lipoprotein-cholesterol; LDL-C, low density lipoprotein-cholesterol; CH, T-Cho/HDL-C; MS, marital status; EL, educational level; FI, family income; HC, hip circumference; WHR, waist–hip ratio; LEE, lower extremity edema; SGOT, serum glutamic-oxaloacetic transaminase; SGPT, serum glutamic-pyruvic transaminase; BMD, bone mass density; RBCs, red blood cells; Hb, hemoglobin; MCV, mean cell volume; UP, urine protein; GU, glucose urine; CS, current smokers; AD, alcohol drinkers; CBN, chewing “betel nut”/“areca catechu”; CKD, chronic kidney disease.

**Table 4:** Model performance in predicting the subjects with MetS and CKD stage 3a or 3b.

Methods	Balanced Accuracy Mean (SD)	Sensitivity Mean (SD)	Specificity Mean (SD)	AUC Mean (SD)
RF	0.698 (0.09)	0.697 (0.21)	0.700 (0.17)	0.657 (0.12)
LGR	0.719 (0.06)	0.678 (0.19)	0.761 (0.25)	0.670 (0.09)
MARS	0.690 (0.07)	0.774 (0.21)	0.606 (0.26)	0.658 (0.09)
XGBoost	0.685 (0.09)	0.615 (0.16)	0.755 (0.16)	0.658 (0.14)
CatBoost	0.710 (0.19)	0.698 (0.17)	0.722 (0.18)	0.667 (0.13)
LightGBM	0.660 (0.12)	0.624 (0.26)	0.697 (0.27)	0.662 (0.14)

RF—random forest; LGR—logistic regression; MARS—multivariate adaptive regression splines; XGBoost—extreme gradient boosting; LightGBM—Light Gradient Boosting Machine; CatBoost—Gradient Boosting with Categorical Features Support.

**Table 5:** DeLong’s test between LGR and the five ML methods of this study.

	RF	MARS	XGBoost	CatBoost	LightGBM
LGR	1.258 (0.208)	1.402 (0.160)	1.615 (0.106)	0.693 (0.488)	0.184 (0.853)

Note: The numbers in parentheses are the corresponding *p*-value; **: *p* < 0.05.

**Table 6:** Overall importance ranking of each predictor variable (only the first 15 variables of Table 2 shown).

Variables	Average Ranking of RF	Average Ranking of LGR	Average Ranking of MARS	Average Ranking of XGBoost	Average Ranking of CatBoost	Average Ranking of LightGBM	Average Ranking of the Six Models (SD)
BMI	14	12	7	12	29	5	13.17 (8.47)
BF	12	14	22	15	20	25	18.00 (5.10)
WC	24	30	26	17	15	26	23.00 (5.80)
SBP	4	4	5	5	2	7	4.50 (1.64)
DBP	25	27	14	29	33	20	24.67 (6.77)
FPG	15	6	11	14	13	14	12.17 (3.31)
L-IOP	20	19	20	23	16	13	18.50 (6.35)
R-IOP	9	2	3	4	8	6	5.33 (6.60)
r-GT	18	37	9	20	22	12	19.67 (9.81)
BUN	1	1	1	1	1	1	1 (0)
UA	22	23	27	16	25	19	22.00 (4.00)
TG	10	33	19	9	7	9	14.50 (9.99)
T-Cho	13	8	23	10	6	15	12.50 (6.09)
HDL-C	7	17	12	8	18	2	10.67 (6.19)
LDL-C	17	16	6	13	28	22	17.00 (7.54)
…	…	…	…	…	…	…	…

## Data Availability

Authorization is required for the use of all data sets collected from the MJ Health Research Foundation. The application procedures are accessed via this link. http://www.mjhrf.org/main/page/release1/en/#release01 (accessed on 1 August 2022).
